# Molecular basis of DEL phenotype in the Chinese population

**DOI:** 10.1186/1471-2350-15-54

**Published:** 2014-05-05

**Authors:** Juan Gu, Xue-Dong Wang, Chao-Peng Shao, Jun Wang, An-Yuan Sun, Li-Hua Huang, Zhao-Lin Pan

**Affiliations:** 1 Department of Clinical Laboratory, The Fifth People’s Hospital of Wuxi, The Clinical College of Nanjing Medical University, Wuxi, Jiangsu 214005, PR China; 2 The Central Laboratory, Wuxi Fifth Affiliated Hospital of Jiangnan University, Wuxi, Jiangsu 214005, PR China; 3 Department of Transfusion, The Second People’s Hospital of Shenzhen, Guangdong 518035, PR China; 4 Department of Clinical Laboratory, The Affiliated Provincial Hospital of Anhui Medical University, Hefei, Anhui 230031, PR China

**Keywords:** Rh blood type, DEL phenotype, *RHD* allele, Chinese population

## Abstract

**Background:**

Rh blood group system is the most complex and immunogenetic blood group system. Prevalent *RHD* alleles vary in different populations. We conducted the present study to examine the genotype of DEL individuals and to elucidate whether novel alleles exist in the Chinese population.

**Methods:**

DEL phenotype was identified by a serologic adsorption-elution method. The nucleotide sequences of ten *RHD* exons and exon-intron boundary regions were evaluated by *RHD* gene-specific PCR-SSP and sequencing.

**Results:**

Of 42306 samples from individual donors and patients, 165 samples were typed as D-negative. Among these D-negative samples, 41 DEL individuals were observed. Thirty-seven DELs were confirmed to have the *RHD*1227A allele. Two DELs seemed to have *RHD-CE-*D hybrid alleles, including one *RHD-CE* (4–7)-D and one *RHD-CE* (2–5)-D. Two novel *RHD* alleles were found among the rest of the DEL samples, including one *RHD*93T > A and one *RHD*838G > A.

**Conclusion:**

In this study, about 24.85% (41/165) of the apparent D-negative Chinese individuals were DEL. *RHD*1227G > A is the most frequent allele in Chinese DEL phenotypes, accounting for 90.24% (37/41). The *RHD-CE*-D hybrid allele might be the second most frequent DEL allele in the Chinese population. Our study would contribute to the understanding of the molecular mechanism underlying D antigen expression of DEL individuals and provide useful information for designing suitable genotyping strategies in RhD-negative individuals in Asia.

## Background

The Rh blood group system is the most complex and immunogenetic blood group system. The Rh antigens are encoded by a pair of homologous genes, *RHD* and *RHCE*, which are located on chromosome 1p34.3-36.1. The two genes have opposite orientations at the *RH* locus, each gene has 10 exons and their sequences are highly homologous. *RHD* encodes the D polypeptide, while *RHCE* gives rise to the C/c and E/e polymorphisms. The *RHD* gene is flanked by an upstream Rh box (at its 5′ end) and a downstream Rh box (at its 3′ end). Both Rh boxes have a length of ≈ 9000 bp in identical orientation and share 98.6% homology. The region (breakpoint region) in which the *RHD* deletion occurs is located within a stretch of 1463 bp in which both Rh boxes have an identical sequence (identity region). The hybrid Rh box, only present when the *RHD* gene is deleted via the proposed mechanism of unequal crossing over, contains a sequence identical to that of the upstream Rh box in the 5′ part preceding the identity region, and the 3′ part following the identity region is identical to the downstream Rh box sequence in European people [1]. Studies of the blood group system have shown that racial differences exist not only in the genetic background of the *RHD* gene but also in the frequencies of the *RHD* allele [2-5]. About 15% of European people are D-negative phenotype and are mostly associated with the deletion of *RHD* between the upstream and the downstream Rhesus boxes. Interestingly, several studies, however, have revealed that there has been the presence of the *RHD* gene in the majority of D-negative Africans and about a quarter of D-negative Africans have an inactive *RHD* gene of pseudogene (*RHD*ψ) with a 37 base pair (bp) insertion and a nonsense mutation [6]. In the Asian population, *RHD*ψ is rare, and a certain percentage of RhD-negative individuals have *RHD-CE*-D^S^ hybrid gene or *RHD*1227A allele. In contrast to the European population, the RhD-negative blood type only occurs in 0.3-0.5% of the Chinese population; [7] however, nearly 30% of the RhD-negative individuals belong to the DEL phenotype, which is a rare variant of the Rh system with a grossly intact *RHD* gene, of which one is the 1227G > A mutation that probably disrupts normal intron splicing. In the European population, the frequency of DEL phenotype was 1:3030 and that of *RHD*1227A allele was 1:9091 [8].

DEL phenotypes derive from several mechanisms. Previous studies showed that DEL phenotype was associated with either a long deletion of *RHD* gene from intron 8 to intron 9, including whole exon 9, or a missense mutation (*RHD*1227G > A) in exon 9, or splice-site mutation, including *RHD* (IVS3 + 1G > A), or a frameshift mutation, including *RHD* (X418 L) or a mutation in the coding region, or premature stop codons [8-13]. Those studies indicate the D-negative trait may be generated by multiple molecular mechanisms.

DEL is the most frequent D variant phenotype in the RhD-negative Chinese population that is confirmed by the adsorption-elute test. Moreover, previous studies through genomic DNA analysis showed that the *RHD*1227 allele is the prevalent causal mutation for DEL individuals in China. Those individuals possess one or two of these alleles with Ccee or CCee phenotypes. Whether there are other DEL alleles in the Chinese population is not yet resolved. The aim of this study was therefore to collect large numbers of blood samples to explore the molecular basis of DEL in the Chinese population.

## Methods

### Blood samples

EDTA-anticoagulated peripheral blood samples were collected from outpatients, inpatient and blood donors at the Anhui Provincial Hospital, the Affiliated Hospital of Anhui Medical University, the Clinical College of Nanjing Medical University, the Affiliated Hospital of Gulin Medical College, the Shaoxing People’s Hospital of Zhejiang Province, the 4^th^ Affiliated Hospital of Jiangsu University, Blood Center of Anhui Province, Shenzhen Blood Center of Guangdong Province and Wuxi Blood Center of Jiangsu Province in the southeast China. This study was approved by the institutional Ethics Review Board of Nanjing Medical University and all subjects provided written informed consent to their participation in it.

### Serological studies

Rh blood type phenotype D status was determined by a microplate technique with IgM monoclonal anti-D reagent (Immucor, Norcross, GA; Gamma Biologicals, Houston, TX) [14]. Subsequently, the Rh C/c and E/e antigens were determined by using agglutinating monoclonal anti-C (Novaclone, Dominion Biologicals, Dartmouth, Canada), anti-E (Gamma Biologicals), anti-c (Immucor) and anti-e reagents (Immucor). All samples that were negative with anti-D in the direct agglutination were retested by using the indirect anti-human globulin test (IAT). The indirect anti-human globulin test was used to detect weak D or partial D phenotype. Furthermore, samples typed as D-negative by the IAT were retested for the DEL phenotype through an adsorption-elution test in tubes. If the result was positive (≥1+), the sample was determined to belong to the DEL phenotype. If the result was negative, the sample was determined to be truly RhD-negative phenotype.

## Molecular studies

### Genomic DNA extraction

Genomic DNA of DEL samples was prepared using QIAamp DNA Blood Mini Kit according to the manufacturer's recommendations (Qiagen, Valencia, CA, USA). The column-bound circulatory DNA was eluted in distilled water and quantified by using a spectrophotometer (NanoDrop 2000c, Thermo Scientific, Wilmington, DE, USA).

### Amplification of *RHD* exons

Primers for *RHD* exons 1 to 10 were given in Table 1. β-actin gene was used as an internal control. Amplification was carried out in a thermal cycler (Veriti 96, Applied Biosystems, Foster City, CA). Cycling conditions for *RHD* exon 1 and 10 were denatured at 95°C for 5 min, then 40 cycles of 30s at 94°C, annealing time was 40 seconds at 64°C (exon 2–4, 6, 8), 62°C (exon 1, 7, 9, 10, *RHD*1227A) and 60°C (exon 5), and extension for 1 min at 72°C. The final extension was at 72°C for 10 minutes. PCR products were visualized by electrophoresis on a 1.5% agarose gel with ethidium bromide staining and these were photographed under UV light.

**Table 1:** Primers used for PCR analysis and DNA sequencing

Primer name*	**Sequence 5′ to 3′**	**Location**^**#**^	**Specificity**	**Product size (bp)**
E1-s	ATGCCTGGTGCTGGTGGA	promoter, -43 to -26	*RHD*/*CE*	228
E1-a	ATTTGCTCCTGTGACCACTT	intron 1, 37 to 18	*RHD*	
E1-seq	ATGCCTGGTGCTGGTGGA			
E2-s	TGACGAGTGAAACTCTATCTCGAT	intron 1, -1064 to -1041	*RHD*	1475
E2-a	GGATTCCTTGTGATACACGGAGTAG	intron 2, 224 to 200	*RHD*	
E2-seq	TGACGAGTGAAACTCTATCTCGAT			
E3-s	GTCGTCCTGGCTCTCCCTCTCT	intron 2, -29 to -8	*RHD*	219
E3-a	CTTTTCTCCCAGGTCCCTCCT	intron 3, 39 to 19	*RHD*/*CE*	
E3-seq	GTCGTCCTGGCTCTCCCTCTCT			
E4-s	GCCGACACTCACTGCTCTTAC	intron 3, -36 to -16	*RHD*/*CE*	378
E4-a	TGAACCTGCTCTGTGAAGTGC	intron 4, 194 to 174	*RHD*	
E4-seq	GCCGACACTCACTGCTCTTAC			
E5-s	TACCTTTGAATTAAGCACTTCACAG	intron 4, -267 to -233	*RHD*	1458
E5-a	TTATTGGCTACTTGGTGCC	intron 5, 1024 to 1006	*RHD*	
E5-seq	TACCTTTGAATTAAGCACTTCACAG			
E6-s	CAGGGTTGCCTTGTTCCCA	intron 5, -95 to -77	*RHD*/*CE*	274
E6-a	CTTCAGCCAAAGCAGAGGAGG	intron 6, 41 to 21	*RHD*	
E6-seq	CAGGGTTGCCTTGTTCCCA			
E7-s	TGCCCATCCCCCTTTGGTGGCC	intron 6, -106 to -85	*RHD*	411
E7-a	CCAAGGTAGGGGCTGGACAG	intron 7, 171 to 152	*RHD*	
E7-seq	TGCCCATCCCCCTTTGGTGGCC			
E8-s	GGTCAGGAGTTCGAGATCAC	intron 7, -594 to -575	*RHD*	709
E8-a	TGGCAATGGTGGAAGAAAG	intron 8, 35 to 16	*RHD*/*CE*	
E8-seq	GGTCAGGAGTTCGAGATCAC			
E9-s	CTGTCGTTTTGACACACAATATTTC	intron 8, -91 to -67	*RHD*	190
E9-a	CACGTTAATAGGTGAAAAATCTTACC	intron 9, 25 to exon 9, 1227	*RHD*	
E9-seq	CTGTCGTTTTGACACACAATATTTC			
E10-s	CAAGAGATCAAGCCAAAATCAGT	intron 9, -67 to -45	*RHD*/*CE*	382
E10-a	AGCTTACTGGATGACCACCA	3’UTR, 291 to 272	*RHD*	
E10-seq	CAAGAGATCAAGCCAAAATCAGT			
*RHD*1227A-s	GATGACCAAGTTTTCTGGAAA	exon 9, 1207 to 1227	*RHD*1227A	109
*RHD*1227A-a	CATAAACAGCAAGTCAACATATATACT	intron9, 88 to 62	*RHD*	
*RHD*1227A-seq	GATGACCAAGTTTTCTGGAAA			
β-actin-s	GGAAATCGTGCGTGACATT	—		473
β-actin-a	CGTCATACTCCTGCTTGCTG	—		

*s = sense primer; a = antisense primer. seq = sequencing primer.

^#^The positions of the synthetic oligonucleotides are indicated relative to their distances from the first nucleotide position of the start codon ATG for all primers in the promoter and in the exons or relative to their adjacent exon-intron boundaries for all other primers.

### PCR-restriction fragment length polymorphism (PCR-RFLP) for *RHD* zygosity determination

The PCR amplification was performed by using the expand high-fidelity PCR system with primers rez7 (consensus, 5′ of the Rh box identity region) and rnb31 (specific for downstream of the Rh box, 3′ of the Rh box identity region) [1]. PCR products were digested with P*st*I at 37°C for 3 h, and the fragments were resolved by electrophoresis on a 1.5% agarose gel with ethidium bromide staining and these were photographed under UV light.

### Purification and sequencing

Amplified DNA products were purified using an isolation kit (NucleoSpin Extract II, Macherey-Nagel, Düren, Germany) and sequenced on a genetic analyzer by DNA technology (ABI 3130 XL, Applied Biosystems, Aarhus, Denmark). The complete *RHD* exons 1 to 10 including adjacent intron regions were sequenced from PCR products with cycle-sequencing kits (BigDye-terminators v.1.1; Applied Biosystems, Weiterstadt, Germany) and Sequence determination of amplicons was performed on both strands using the respective PCR primers with an ABI PRISM 3730 automated sequencer (Applied Biosystems, Foster City, USA). The nucleotide and deduced amino acid sequences were analyzed and compared with the published sequences using computer software (Lasergene 99, DNASTAR, Madison, WI) as a sequencing analysis tool. All sequenced PCR products were compared with GenBank Accession Number BN000065.

## Results

### Serologic studies

A total of 165 apparent D-negative samples were found among the 42306 blood donors and patients through the microplate determination. When the D-negative samples were retested by the IAT and the adsorption-elution test, a total of 41 DELs were found among these samples. In the Chinese population examined, approximately 24.58% (41/165) of the apparent D-negative individuals belong to DEL phenotypes (Table 2).

**Table 2:** Results of phenotype and genotype analyses of the 41 DEL samples

**Sample no.**	**Number of subjects**	**RhD**			**RhCcEe**	***RHD *** zygosity*	***RHD *** **allele**
		**Micro-plate**	**IAT**	**Adsorption/elute**			
13001-13030	30	–	–	+	Ccee	*RHD*+/*RHD-*	*RHD*1227A
13031-13033	3	–	–	+	CCee	*RHD*+/*RHD-*	*RHD*1227A
13034-13035	2	–	–	+	Ccee	*RHD*+/*RHD+*	*RHD*1227A
13036	1	–	–	+	CCEe	*RHD*+/*RHD-*	*RHD*1227A
13037	1	–	–	+	CcEe	*RHD*+/*RHD-*	*RHD*1227A
13038	1	–	–	+	Ccee	*RHD*+/*RHD-*	*RHD*-*CE* (2-5)-D
13039	1	–	–	+	Ccee	*RHD*+/*RHD-*	*RHD*-CE (4-7)-D
13040	1	–	–	+	Ccee	*RHD*+/*RHD+*	*RHD*93T>A
13041	1	–	–	+	Ccee	*RHD*+/*RHD-*	*RHD*838G>A

*Presence (+) or absence (–) of the *RHD* gene.

### Molecular characterization of DELs

**Figure 1:**
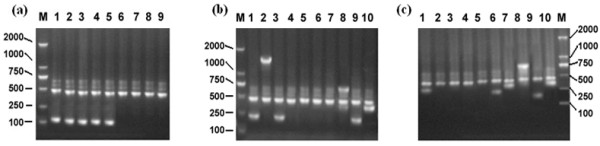
**Results of the PCR-SSP analysis.** In all lanes, a 473-bp product was amplified as the internal positive control; M, molecular marker (2000, 1000, 750, 500, 250 and 100 bp, respectively). **(a)** PCR-SSP for *RHD*1227A allele in 9 DEL samples. Lanes 1 to 9 showed the PCR-SSP results of 9 DEL samples. Lanes 1 to 5 showed that the *RHD*1227A specific amplifications (band of 109 bp) were positive (Sample No.13001-13005). They were shown as representatives of 37 DEL individuals carrying *RHD*1227A allele. Lane 6 to 9 showed that the *RHD*1227A specific amplifications were negative (Sample No.13038-13041). **(b)** PCR-SSP for *RHD-CE* (4–7)-D in one DEL sample (Sample No.13039). Lanes 1 to 10 showed the PCR-SSP results of the *RHD* exons 1 to 10; Lanes 4 to 7 showed that the *RHD* specific amplifications were negative for exons 4 to 7; *RHD* specific amplifications were positive for exons 1 to 3 and 8 to 10. **(c)** PCR-SSP for *RHD-CE* (2–5)-D in one DEL sample (Sample No.13038); lanes 1 to 10 showed the PCR-SSP results of the *RHD* exons 1 to 10; Lanes 2 to 5 showed that the *RHD* specific amplifications were negative for exons 2 to 5; *RHD* specific amplifications were positive for exons 1, 6 to 10.

**Figure 2:**
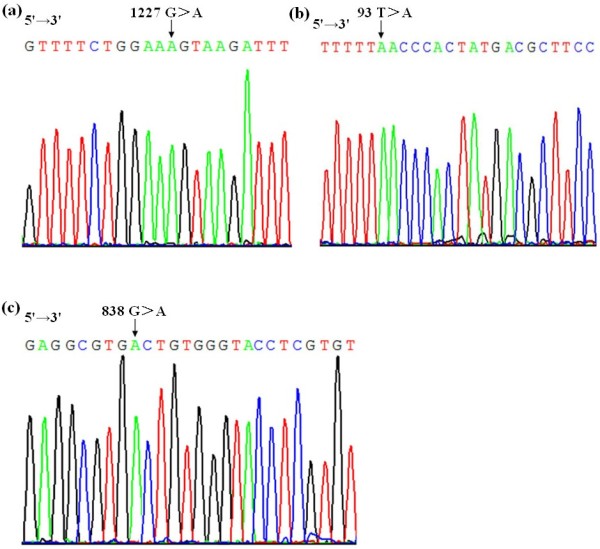
**Electropherograms of *RHD *DNA sequencing. (a)** Sequencing analysis of the *RHD*1227A allele. The arrow indicates the position of nucleotide mutation between *RHD* exon 9 and intron 9. A representative example of 37 *RHD*1227A genotyped cases is shown; **(b)** Sequencing analysis of the *RHD*93T > A allele in one DEL sample (Sample No.13040). The arrow indicates the position of nucleotide mutation in *RHD* exon 1; **(c)** Sequencing analysis of the *RHD*838G > A allele in one DEL sample (Sample No.13041). The arrow indicates the position of nucleotide mutation in *RHD* exon 6.

Among the 41 DELs samples, a total of 37 samples were determined to have the *RHD*1227A allele by PCR-SSP analysis and sequencing (Table 2, Figures 1a and 2a), accounting for 90.24% (37/41) in the Chinese DEL individuals. All of these individuals carrying *RHD*1227A allele were demonstrated that the mutation is located in the splice site at the exon 9 and intron 9 junctions. The remaining four DEL samples did not have *RHD*1227A (Figure 1a; lines 6 to 9). Two samples seemed to have *RHD-CE*-D hybrid alleles. According to the PCR-SSP for the *RHD* gene exons 1 to 10 determination, the first sample (sample No: 13039) lacked *RHD* exons 4 to7, the second sample (sample No: 13038) lacked exons 2 to 5 (Figure 1b and c). The two samples were confirmed to be *RHD-CE* (4–7)-D and *RHD-CE* (2–5)-D, respectively. No mutation was found in the *RHD*-specific exons by sequencing.

Two novel *RHD* alleles were found in the remaining two DEL samples. One sample (sample No: 13040) was found to have a *RHD*93T > A mutation (Figure 2b), the other sample (sample No: 13041) had a *RHD*838G > A mutation (Figure 2c). Their DNA sequences were deposited under GenBank accession numbers: *RHD*93T > A (KJ558352 for exon 1), *RHD*838G > A (KJ558353 for exon 6). The *RHD*93T > A and *RHD*838G > A alleles were missense mutations, which caused F31L and A280T amino acid mutations, respectively, both of the two mutations occurred in the RhD transmembrane domain. The samples with *RHD*93T > A and *RHD*838G > A alleles belong to Ccee phenotype. The sample assigned to *RHD*93T > A allele was found to be *RHD*+/*RHD* + homozygote by PCR-RFLP analysis and the other sample with *RHD*838G > A allele was *RHD*+/*RHD*– heterozygote (Table 2).

## Discussion

DEL phenotype was determined on the basis of non-agglutination by the IAT procedure and positive result by an adsorption-elution technique. Molecular studies have shown that a heterogeneous array of variant *RHD* allele can result in the DEL phenotype [15]. Körmöczi and colleagues suggested that the DEL phenotypes might be subdivided into two groups, partial DEL with characteristic D epitope loss caused by either *RHD-CE*-D hybrid genes or *RHD* point mutation such as carrier of *RHD* (IVS3 + 1G > A) affecting extracellular RhD loops and complete DEL where the majority of D epitopes are conserved such as *RHD*1227A [16]. According to the published data [9,17] and our results, the *RHD*1227A allele was the most frequent allele in the Chinese DEL individuals. In our study, two DELs seemed to have *RHD-CE-*D hybrid alleles, including one *RHD-CE* (4–7)-D and one *RHD-CE* (2–5)-D. The *RHD-CE* (4–7)-D was thought to be a DEL allele by our laboratory, while a similar hybrid *RHD-CE* (4–7)-D was described as D-negative [10]. Whether there were miniscule differences between these two alleles has not yet been clarified. The *RHD-CE* (2–5)-D was similar to the *RH* allele that is also called D^VI^ type 4. Hasekura *et al*. have suggested that some DELs were caused by a trans effect of C on a haplotype containing a weak D allele [18]. Whether the D^VI^-like DEL has a similar mechanism, that the DEL was resulted from the influence of C in trans on a haplotype with a partial D allele, needs to be further analysed. The hybrid gene individuals were detected with Rh box zygosity. They were found to be *RHD*+/*RHD*– heterozygote (Table 2). These results may also be in concordance with the studies of Li et al [17].

Two samples with the novel *RHD*93T > A and *RHD*838G > A alleles showed slightly stronger positive results (2+) than other DEL samples (1+) in the adsorption-elution test. This phenomenon suggested that there might be scant differences in the D antigen between these two samples and other DELs. The possibility that these two samples might be two new kinds of weak D^S^ could not be excluded.

DEL is the weakest D positive phenotype, whether the potential danger that DEL RBCs might cause a clinical transfusion reaction cannot be completely excluded. Recipients with a truly D-negative phenotype developed anti-D after transfusion with DEL RBCs [19,20]. Recently, Richard and his colleagues found a patient with a DEL phenotype who developed anti-D [11,12]. Other members of our team have not yet reported analogous cases in the Chinese population. Shao and his colleagues found that the Asian type DEL displays the complete repertoire of RhD antigen epitopes. They suggested that people in East Asia who carry the DEL variants could safely receive blood transfusion from RhD-positive donors [21]. Our previous study also supported the biochemical observations that DEL variants express normal RhD and pose virtually no risk of inducing anti-D antibodies [22]. We also confirmed that this is an exact model test that people carrying the DEL variants can safely receive blood transfusion from RhD-positive donors.

Throughout the world, the majority of DEL phenotypes are misinterpreted as D-negative owing to the limits of routine typing. For this reason, the recipient with a truly D-negative phenotype would be likely to develop into anti-D alloimmunization after transfusion with DEL RBCs. So it would be of interest to routinely screen serologically D-negative donors for the presence of the *RHD* gene in order to discriminate all clinically relevant *RHD* DEL alleles. An optimized PCR strategy that checks for *RHD*-specific polymorphism, supplemented by the specific detection of aberrant alleles, would be a good choice. Samples carrying *RHD* deletion should be correctly typed by multiple PCR utilized for *RHD* genotyping. Testing additional *RHD* exons would improve the specificity of RhD prediction. Although direct sequencing is currently the gold standard in mutation identification, it is still relatively laborious and expensive, whereas PCR-SSP as a one-step method is less expensive and less time consuming. In any case, the combined use of serologic and molecular D typing techniques may reduce the number of such transfusion incidents. All of these findings suggested that it might be important to explore the molecular basis of DEL individuals in Asia and to form molecular screening techniques to determine the DEL phenotype accurately.

## Conclusion

In this study, about 24.85% (41/165) of the apparent D-negative Chinese individuals were DEL. *RHD*1227G > A is the most frequent allele in Chinese DEL phenotypes, accounting for 90.24% (37/41). The *RHD-CE*-D hybrid allele might be the second most frequent DEL allele in the Chinese population. Novel DEL alleles are still relatively rare and frequencies of occurrence are also very low. Our study would contribute to the understanding of the molecular mechanism underlying D antigen expression of DEL individuals and provide useful information for designing suitable genotyping and transfusion strategies for the RhD-negative individuals in Asia.

## Competing interests

The authors declare that they have no competing interests.

## Authors’ contributions

Authors who obtained funding included XDW and JG; Authors who participated in conception and design were XDW, JG and CPS; Authors who carried out data analysis and interpretation included JG, AYS, JW, ZLP and LHH; Authors who drafted and finalized the manuscript were XDW, JG, and CPS. All authors read and approved the final manuscript.

## Pre-publication history

The pre-publication history for this paper can be accessed here:

http://www.biomedcentral.com/1471-2350/15/54/prepub
